# The Role of Surface Topography on Deformation-Induced Magnetization under Inhomogeneous Elastic-Plastic Deformation

**DOI:** 10.3390/ma11091518

**Published:** 2018-08-23

**Authors:** Nadja Sonntag, Birgit Skrotzki, Robert Stegemann, Peter Löwe, Marc Kreutzbruck

**Affiliations:** 1 Bundesanstalt für Materialforschung und -prüfung (BAM), Department 5: Materials Engineering, Unter den Eichen 87, 12205 Berlin, Germany; nadja.sonntag@bam.de (N.S.); peter.loewe@bam.de (P.L.); 2 Bundesanstalt für Materialforschung und -prüfung (BAM), Department 8: Non-Destructive Testing, Unter den Eichen 87, 12205 Berlin, Germany; robert.stegemann@bam.de; 3 University of Stuttgart, IKT, Pfaffenwaldring 32, 70569 Stuttgart, Germany; marc.kreutzbruck@ikt.uni-stuttgart.de

**Keywords:** magnetic stray fields, magnetomechanical effect, damage, topography, multiaxial deformation, notch, plastic deformation, metal magnetic memory, digital image correlation, structural steel

## Abstract

It is widely accepted that the magnetic state of a ferromagnetic material may be irreversibly altered by mechanical loading due to magnetoelastic effects. A novel standardized nondestructive testing (NDT) technique uses weak magnetic stray fields, which are assumed to arise from inhomogeneous deformation, for structural health monitoring (i.e., for detection and assessment of damage). However, the mechanical and microstructural complexity of damage has hitherto only been insufficiently considered. The aim of this study is to discuss the phenomenon of inhomogeneous “self-magnetization” of a polycrystalline ferromagnetic material under inhomogeneous deformation experimentally and with stronger material-mechanical focus. To this end, notched specimens were elastically and plastically deformed. Surface magnetic states were measured by a three-axis giant magnetoresistant (GMR) sensor and were compared with strain field (digital image correlation) and optical topography measurements. It is demonstrated that the stray fields do not solely form due to magnetoelastic effects. Instead, inhomogeneous plastic deformation causes topography, which is one of the main origins for the magnetic stray field formation. Additionally, if not considered, topography may falsify the magnetic signals due to variable lift-off values. The correlation of magnetic vector components with mechanical tensors, particularly for multiaxial stress/strain states and inhomogeneous elastic-plastic deformations remains an issue.

## 1. Introduction

The Villari (or magnetoelastic) effect [1] (i.e., the change in the magnetic state of a ferromagnetic material due to uniaxial, elastic loading) is widely accepted. For example, magnetoelastic interactions are utilized in force and torque measurements using magnetoelastic sensors [2]. It is also known that short- or long-range distortion fields of single or multiple dislocations interact with the magnetic microstructure (magnetic domains) [3,4]. Consequently, plastic deformation can also induce changes in microscopic and macroscopic magnetic properties such as in magnetic permeability [5,6]. These magnetomechanical effects have been widely described for homogeneous stress and strain states [7,8,9,10,11,12]. Over the last 20 years, a nondestructive testing (NDT) technique, called the metal magnetic memory (MMM) method, has been established, which is suggested for the detection and assessment of “early damage” on the basis of natural, stress-induced magnetic stray fields [13,14,15,16,17,18,19,20,21,22,23,24]. However, the mechanical and microstructural complexity of inhomogeneous deformation and damage has so far been insufficiently considered in the MMM literature. 

**Figure 1:**
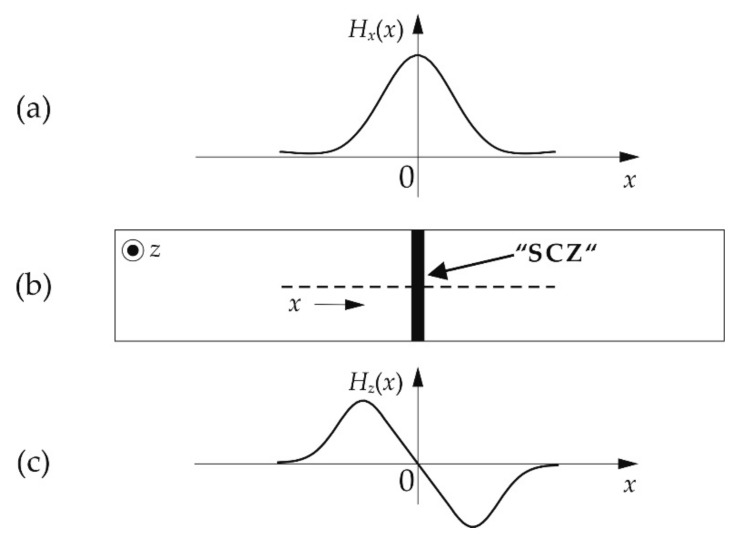
Schematic representation of typical MMM signals; adapted from [26], Copyright (2010), with kind permission from Elsevier: (**a**) in the presence of a “stress concentration zone” (SCZ, (**b**)), the longitudinal in-plane component *H_x_*(*x*) exhibits a peak, while (**c**) the normal field component *H_z_*(*x*) reverses its direction.

The metal magnetic memory technique is a passive-magnetic, standardized nondestructive testing (NDT) method [25], which is proposed for the detection of so-called “stress concentration zones” (SCZ) [18,19,24], as well as for damage assessment [13,14,26] of ferromagnetic materials and components. It is based on the assumption that localized deformations or (macroscopic) mechanical stress gradients involve localized magnetomechanical interactions. Due to this inhomogeneous magnetization process, weak macroscopic magnetic stray fields develop (cf. Figure 1), which are referred to as either self-magnetic leakage fields (SMLF) [23,25,27] or residual magnetic fields (RMF) [13,28,29] in the MMM literature. In contrast to conventional electromagnetic NDT techniques (such as magnetic flux leakage testing), an active magnetization of the test object (e.g., by coils or permanent magnets) is not required for the stray field detection. 

The MMM technique has gained considerable interest because of its simplicity. The formation of deformation-induced magnetic stray fields is also an interesting phenomenon from a materials science point of view. However, the underlying microstructural and mechanical origins of the magnetic stray field formation under inhomogeneous elastic-plastic deformation are still not fully understood. Additionally, the magnetomechanical hypotheses were not validated experimentally: it is assumed that the stray fields occur “on the components’ surfaces in the zones of stable slip bands of dislocations under the exposure to operational and residual stresses …” [22]. Yet, the literature in the field of MMM neither explains nor experimentally verifies, how the observed macroscopic stray fields correlate with pinning of magnetic domain walls by dislocations in randomly oriented grains and solely under the excitation of the Earth’s magnetic field. Correlations of magnetic stray fields with experimentally determined dislocation densities are also missing. Apart from lacking microstructural studies, mechanical aspects of damage are not adequately considered.

Finally, it should be noted that magnetoelastic or magnetomechanical models and theories [7,8,9,10,11,30], which MMM authors refer to, have once been developed for uniaxial and homogeneous stress distributions in the material. However, damage and localized inhomogeneous deformation processes (as common in components) provoke complex states of stress and strain in the material that cannot be determined solely from (applied) nominal stress values. In other words, mechanical gradients arise and the actual stress and strain values vary depending on location. Therefore, correlations between local magnetic field strengths and nominal (applied) stresses such as performed in [13,16,17,18,20,23] appear questionable. In addition, the directionality of magnetic (vector) components and mechanical tensors (and their components) is also hardly considered. For example, some authors propose considering the magnetic field components acting parallel to the surface (hereafter referred to as in-plane components *H_x_* and *H_y_*) for correlations with the stress state, e.g., in [13,28,31,32,33], while others suggest analyzing the normal field component perpendicular to the surface, e.g., in [16,19,34] (hereafter referred to as *H_z_*). Moreover, inhomogeneous plastic deformation (e.g., during necking in tensile testing) causes surface topographies, whose significance has so far not been recognized. 

Therefore, the aim of this study is to systematically investigate the phenomenon of inhomogeneous, deformation-induced magnetization of a ductile, polycrystalline ferromagnetic material under localized elastic-plastic deformation experimentally and with a stronger material-mechanical focus. For this purpose, a common ferromagnetic structural steel (S235JR) was investigated by monotonic tensile testing of notched test pieces to study the magnetic response under elastic and plastic multiaxial loading conditions.

## 2. Materials and Methods

### 2.1. Material

The hot-rolled low-carbon structural steel S235JR with a ferritic-pearlitic microstructure and an average grain diameter of approx. 15 µm was chosen to represent a moderate strength technical steel with medium soft-magnetic behavior. Its chemical composition and its mechanical properties are given in Table 1 and Table 2.

### 2.2. Specimen Geometry and Preparation

**Figure 2:**
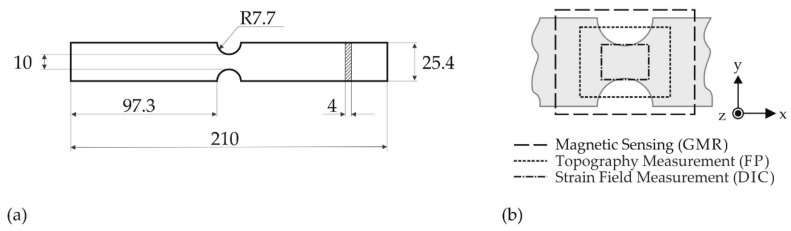
(**a**) Tensile specimen geometry with dimensions (in mm); and (**b**) notched specimen region (grey) and schematic representation of different measurement regions discussed.

Flat tensile specimens were fabricated from a 6.0 mm thick sheet with two opposing semi-circular notches as depicted in Figure 2a. The notches create a multiaxial stress state during mechanical loading. They enlarge the inhomogeneously deformed specimen region as compared to natural necks and enable the observation of inhomogeneous magnetic field distributions right from the onset of yielding up to the very advanced loading stages. The elastic stress concentration factor *K*_t_ = 1.4 for the given geometry in axial tension was determined based on Roark’s formulas [35]. 

The assessed nominal yield stress $\sigma_{y}^{n}$ for the notched geometry (cf. Figure 2a) is determined by *K*_t_ and the upper yield strength, *R_eH_* (Table 2):

(1) $$\sigma_{y}^{n}=\frac{R_{eH}}{K_{t}}=183\;MPa$$

To preclude any magnetic perturbation due to a fluctuating chemical composition and pronounced surface roughness, the oxide scale had been fully removed by grinding. Further, all specimens were demagnetized in a decreasing alternating field after fabrication and prior to mechanical loading. This state is referred to as the “as-received state”.

Depending on the respective examination method described in Section 2.4, Section 2.5 and Section 2.6, the discussed measurement areas vary (Figure 2b). Nevertheless, all of them cover the most severely deformed surface region. Directions of specimen length, width, and thickness are referred to as *x*, *y*, and *z* directions as given in Figure 2b.

### 2.3. Mechanical Loading

All specimens were deformed in monotonic tension using a 100 kN electro-mechanical testing machine (Instron 8562, Instron, Norwood, MA, USA). Loading was performed in the *x* direction at room temperature in position control and with a constant crosshead separation rate of 0.675 mm/min. Tests were stopped after different amounts of strain (Table 3) covering the elastic and plastic regions of the stress-strain-curve. To prevent the specimens from artificial magnetization by the extensometers, nominal strains were recorded on an additional specimen, tested in the same set-up, by two opposing axial extensometers with an initial gauge length of 25 mm. The nominal strain values of specimens N1 to N5 (Table 3) were obtained from the experimentally-determined relationship between piston displacement and extensometer strains. 

### 2.4. Magnetic Sensing

Magnetic fields of the as-received and of the deformed specimen surfaces were detected by a three-axis GMR (giant magnetoresistance) spin valve magnetometer (prototype GA757B, Sensitec, Lahnau, Germany) [36]. This sensor is characterized by a higher spatial resolution as compared to commercial MMM testers (fluxgate magnetometers). It contains specifically-arranged magnetic flux concentrators [37], which allow a simultaneous and Cartesian detection (cf. coordinate system in Figure 2b) of the normal (*H_z_*) and in-plane (*H_x_*, *H_y_*) magnetic field components. Two bar-shaped flux concentrators sensitive to *H_z_* have a longitudinal section of 160 µm × 10 μm, which affects the normal component of the magnetic field to be measured by 90° to the sensitive direction of the GMR elements. The flux concentrators for the in-plane directions *H_x_* and *H_y_* are semicircular structures with a diameter of 140 μm. The sensitivity in the linear range of the sensor is about 16 mV/V/kA/m for the *z* component and about 80 mV/V/kA/m for the *x* and *y* components. Fundamentals of the magnetic field measurement by GMR solid state sensors can be found elsewhere [38,39]. 

Magnetic field matrices of the *x*-*y* surfaces were recorded by an automated scanning process with an equidistant measuring point distance (Δ*x*) of 16 µm, a line distance (Δ*y*) of 141 µm and a constant sensor height, *D*. The sensor height *D* was set to 600 µm for each specimen at the highest point of the *x*-*y* surface, which resulted in a spatial resolution of approx. 200 µm [37]. Individual magnetic profiles were offset-corrected; the original data are provided in Appendix A. Additional information on offset-correction and specimen positioning, e.g., with respect to the Earth’s magnetic field, is found in Appendix B.

### 2.5. Strain Field Measurements

To obtain spatially-resolved information about strains and strain gradients at the deformed specimen surfaces, optical strain field measurements were conducted by digital image correlation (DIC) on an additional test piece, N_DIC_, under the same mechanical testing conditions: a high contrast stochastic grey scale pattern had been sprayed onto the specimen surface prior to mechanical loading. N_DIC_ was then deformed until fracture, while 50 photographs of the notched sample region, sized 18.5 mm × 12.3 mm (Figure 2b), were taken time-triggered by a high resolution optical camera system. The computational strain calculation was realized by ARAMIS 6.3.1 (GOM, Braunschweig, Germany) software. The basic principle of digital image correlation is described elsewhere [40]. The two-dimensional strain fields were afterwards assigned to the corresponding deformation states of specimens N1 to N5 considering final position, load and time values. These reference strain fields are referred to as N1^Ref^ to N5^Ref^.

### 2.6. Topography Measurements

Surface topographies of the deformed samples (N1 to N5) were optically measured by fringe projection (FP) technique in a measuring region of 24 mm × 18 mm (Figure 2b) after mechanical loading. A 3D structured light scanner (MikroCAD compact, GFMesstechnik, Teltow, Germany) with a lateral resolution of 2 µm and a vertical resolution of 100 nm was used. FP profilometry is based on the observation that a regular stripe pattern projected onto an uneven three-dimensional object appears bend from a tilted viewing angle. As the regular patterns are projected, images of the distorted fringe patterns are captured from a known triangulation angle. The topography-induced phase modulations of the fringes are calculated, and an unwrapping algorithm is usually applied to reconstruct the height coordinates, *z* of the specimen surfaces. Further information on the measuring principle is found for instance in [41]. 

Additionally, some topographic details were captured by white-light interference microscopy (WLIM) using a NewView 5022 multi probe set-up (ZYGO, Middlefield, OH, USA) with a lateral resolution of 120 nm and a vertical resolution of 0.1 nm. In white-light interferometry, a beam splitter divides a white-light beam coming from a light source into a reference beam and a measurement beam. The former meets a reference mirror, the latter is incident on the sample surface. During the measurement, the distance between sample and interferometer is varied over a *z* displacement unit and interference signals of the two reflected recombined beams are usually detected by a charge-coupled device (CCD) camera. For each point of the sample surface, optical interference occurs when the path lengths of the reflected measurement and reference beams are equal. Thus, the interference signals being dependent on the distance between the sample and the interferometer can be used to infer the height coordinates of the surfaces under investigation. The measuring principle is described in more detail, for example, in [42].

### 2.7. Experimental Sequence

The general experimental procedure after demagnetization (as-received state) was: (1) magnetic sensing of the as-received surfaces; (2) mounting to the test machine; (3) mechanical loading; (4) dismounting; (5) magnetic sensing of the deformed surfaces; (6) topography measurements; and (7) assignment of the corresponding reference strain fields, N^Ref^. This sequence was applied to all specimens (N1 to N5).

## 3. Results

### 3.1. Mechanical Loading

**Figure 3:**
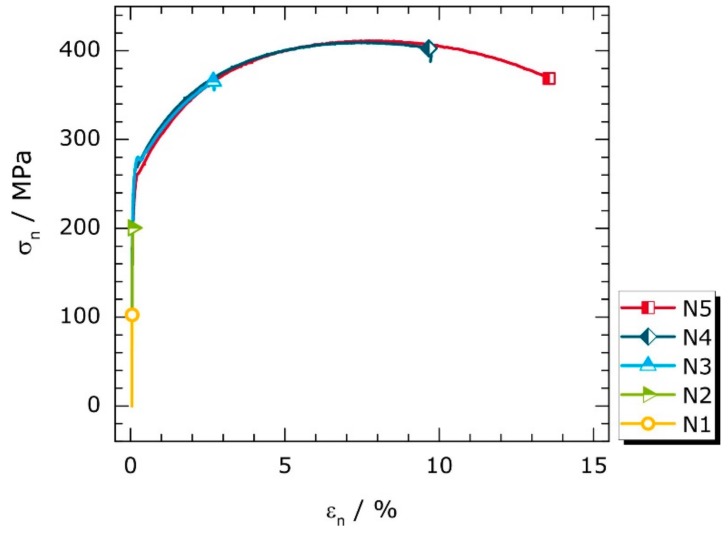
Nominal stress-nominal strain curves of specimens N1 to N5; symbols indicate deformation limits.

Figure 3 shows the nominal stress-nominal strain curves of the deformed, notched specimens N1 to N5 as a result of tensile testing. N1 to N5 were deformed to different nominal strains. Since the curves are almost identical in early loading stages, the deformation limits are additionally indicated by symbols. The colors assigned to the deformation states of N1 to N5 will be retained in subsequent sections. 

Owing to the stress raising effect of the notches, local small-scale plastic yielding is already initiated at stress values below the upper yield strength, *R*_eH_ (256 MPa). The nominal yield stress for the notched geometry ($\sigma_{y}^{n}$) was determined to be 183 MPa using Equation (1). Therefore, microplastic deformation is expected to occur first in specimen N2 in the notched region despite its curve linearity seen in Figure 3. Since localized plastic flow and the formation of Lüders bands are largely concentrated on the notched specimen regions, the typical yield point phenomenon of unalloyed low-carbon steels [43] is not easily recognized in the nominal stress-strain curves. Once the entire cross-section is plasticized, i.e., the fully plastic state is attained (N3 to N5), plastic deformation processes can easily be identified by nonlinear curve progressions. 

### 3.2. Deformation-Induced Magnetic Stray Fields

#### 3.2.1. Analysis of Individual Magnetic Field Profiles

**Figure 4:**
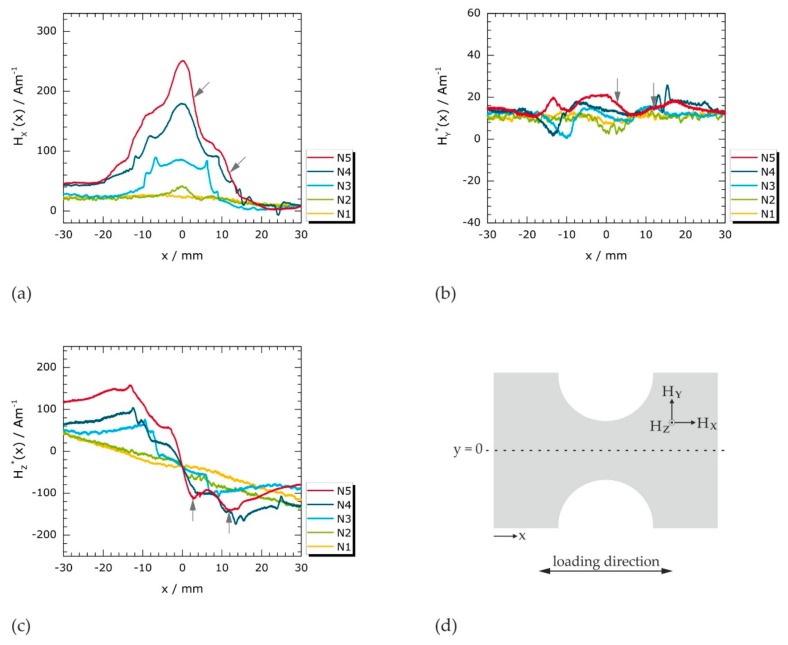
Magnetic field profiles of deformed specimens obtained from giant magnetoresistance (GMR) measurements and extracted from GMR scans (cf. Figure 5) for *y* = 0 (along the dotted line in (**d**)): (**a**) longitudinal in-plane component H_x_*(*x*); (**b**) transverse in-plane component H_y_*(*x*); and (**c**) normal component H_z_*(*x*).

**Figure 5:**
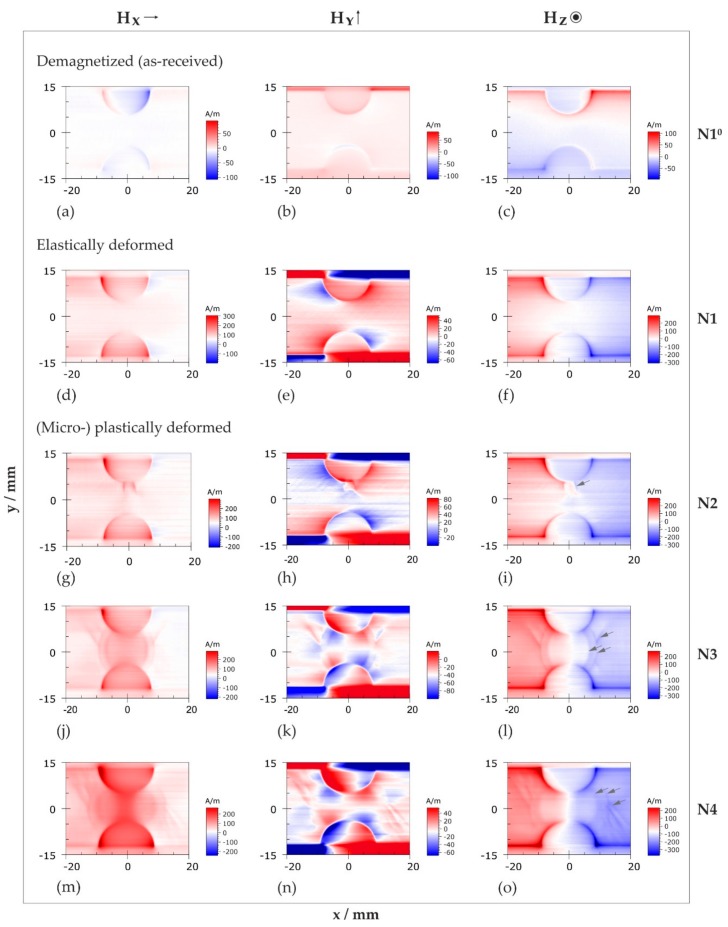
Magnetic stray fields in notched specimen regions before and after mechanical loading obtained from giant magnetoresistance (GMR) measurements. Cartesian components of magnetic field vector, *H_x_*, *H_y_*, and *H_z_*, are arranged in columns; their directions with respect to the specimen coordinate system are indicated at the top. Each row provides the magnetic information of a specific deformation state (N1 to N4): (**a**–**c**) as-received state; (**d**–**f**) elastically deformed state; (**g**–**o**) plastically deformed states. Different intensity scales of the diagrams were chosen for optimum resolution.

Discussions and interpretations of magnetic stray fields caused by concentrated deformation processes are usually based on the analysis of individual profile lines in MMM testing [13,14,17,18,26]. Therefore, profiles of the Cartesian magnetic field components are analyzed first (Figure 4a–c). They were extracted from the magnetic field matrices presented in Section 3.2.2 (cf. Figure 5) along a horizontal line (Figure 4d) for each specific loading case (N1 to N5). The data shown in Figure 4 were offset-corrected as described in Appendix B, which is indicated by asterisks (H_x_*, Hy*, Hz*). The original data (*H_x_*, *H_y_*, *H_z_*) is provided in Appendix A.

The measured magnetic fields are analyzed as functions of the *x* direction along *y* = 0 (Figure 4d) for two reasons: first, tensile stresses create an additional magnetoelastic (stress) anisotropy [12,44,45] and quasi-isotropic materials with positive magnetostriction are favorably polarized along the tensile direction [46]. Since the tensile load is applied in *x* direction and the specimens are, thus, mainly strained longitudinally, pronounced changes in magnetic polarization can be expected in this direction. Second, disturbances of the magnetic signals by the sample edges and, particularly, by the notches are minimized along the selected horizontal line, *y* = 0.

The H_x_*(*x*) profiles of specimens N1 to N5 are presented in Figure 4a. While H_x_*(*x*) is approximately constant after elastic deformation (N1), characteristic peaks are found after (micro) plastic deformation (N2 to N5). The peak maxima appear close to the center, at *x* ≈ 0. Once localized plastic deformation is initiated, both peak amplitude and full width at half maximum (*FWHM*) of the depicted H_x_*(*x*) profiles systematically rise with increasing deformation (N2 to N5).

Systematic changes may also be recognized in the H_z_*(*x*) profiles presented in Figure 4c: with increasing applied load, an increase of both the absolute field values and the curve slopes in the central region is observed. In contrast to the in-plane component H_x_*(*x*) (Figure 4a) exhibiting global maxima at *x* ≈ 0, the normal component H_z_*(*x*) changes its direction with field values being larger than the Earth’s magnetic field for *x* > 0 and being lower for *x* < 0. The qualitative features of H_x_* and H_z_* are consistent with those reported in the MMM literature (cf. Figure 1) [17,18,26,29].

However, neither pronounced peaks nor slope changes indicate an interrelation between the transverse in-plane component H_y_* (Figure 4b) and the degree of deformation. Instead, all H_y_*(*x*) profiles show only slight deviations from otherwise almost constant curve progressions. Note that the field range of H_y_*(*x*) is approximately ten times lower than observed for H_x_*(*x*) and H_z_*(*x*).

It is clearly seen in Figure 4a–c that the Cartesian magnetic vector components H_x_*, H_y_*, and H_z_* provide directional information. This directionality causes specific qualitative and quantitative differences in their (global) signal appearances. However, in addition to their divergent global signal trends, several local discontinuities are found in H_x_*(*x*), H_y_*(*x*), and H_z_*(*x*) at the same *x* positions for each respective plastic deformation state. At these positions, which are highlighted by arrows in Figure 4a–c, H_z_* exhibits local maxima that correspond to local inflection points in H_x_* and H_y_*.

The physical causes of these local discontinuities (at very specific positions) are unlikely to be clarified solely by the analysis of individual magnetic field profiles. Therefore, the *H_x_*, *H_y_*, and *H_z_* distributions within the entire notched *x*-*y* sample regions are examined in Section 3.2.2.

#### 3.2.2. Two-Dimensional Representation of Magnetic Field Distributions

Figure 5 displays the distributions of the magnetic field components *H_x_*, *H_y_*, and *H_z_*, which were determined in the notched sample regions before and after mechanical loading by conducting an automated scanning process: the individual magnetic field components are arranged in columns, while each row contains the magnetic information of a specific deformation state. The directions of the *H_x_*, *H_y_*, and *H_z_* with respect to the sample coordinate system are indicated above the respective columns. In contrast to the profiles discussed in Section 3.2.1, Figure 5 provides the original data, whereby the color scales of the deformed states (Figure 5d–o) are selected according to their specific offset values. In other words, red and blue colors in Figure 5d–o correspond to magnetic field strengths that are supposed to be larger (positive values) and smaller (negative values), respectively, than the respective Earth’s magnetic field component. Due to magnetic edge effects, the sample contours are indicated in all depicted sub-images. Therefore, a sufficient spatial allocation of the signals is possible, albeit the actual dimensions of the notched regions are most likely to be extracted from the normal *H_z_* component.

##### As-Received State and Elastic Deformation

The as-received magnetic state is represented in Figure 5a–c using the example of specimen N1 before loading (designated N1^0^): the magnetic field distribution in the measurement region can be considered as approximately homogeneous. The absolute field values of the two in-plane components *H_x_* and *H_y_* are in the order of only a few A/m and, thus, negligibly small (Figure 5a,b). A small residual magnetization in the transverse direction (*y* direction) after the conventional demagnetization process is detectable in the normal component *H_z_*, where peak values of less than ±100 A/m occur at the sample edges (Figure 5c).

Loading specimen N1 within the elastic range changes the as-received magnetic state irreversibly (Figure 5d–f): while the magnetic field distribution of the in-plane component *H_x_* remains homogeneous (pale red color in Figure 5d) and magnetic field gradients are hardly perceptible, four regions with alternating field directions (positive (red) and negative (blue) field values, respectively) emerge in the *H_y_* component (Figure 5e). 

These extrema are separated by smooth transitions along the horizontal (*y* = 0) and vertical (*x* = 0) symmetry axes and exhibit extreme values close to the notched specimen edges. In contrast to the as-received state, the *H_z_* distribution clearly indicates a longitudinal magnetization (in the *x* direction) after elastic deformation (Figure 5f); two large extrema are formed left (red) and right (blue) of the vertical symmetry axis, whereby the magnetic field direction smoothly reverses in a wide area between the notches (white color).

##### Plastic Deformation

The magnetic field distributions of three plastically deformed specimen surfaces (N2 to N4) are depicted in Figure 5g–o. The global characteristics of *H_x_*, *H_y_* and *H_z_* are very similar to those observed after elastic deformation: predominantly positive (red) field values are observed in the *H_x_* component; *H_y_* changes its direction several times (alternating red/blue regions) and *H_z_* retains two large opposing stray field components on either side of the vertical symmetry axis.

The gradual magnetization of the specimens in *x* direction is revealed by systematic quantitative changes in the *H_x_* component (Figure 5g,j,m), i.e., *H_x_* is successively rising with ascending applied mechanical load, whereby the maximum field values occur in a defined region between the notches. In contrast, no significant changes are observed at greater distances (e.g., at *x* = 20 mm). Thanks to the two-dimensional data representation, first magnetic discontinuities resulting from microplastic deformation are disclosed in the upper notch root region of N2 (Figure 5g). In the more severely deformed specimens (Figure 5j,m), this magnetically distorted region covers the whole cross-section of the notched zone and expands in the *x* direction with increasing applied load (specimen N5 is not included here since it adds no additional value).

The magnetization in longitudinal direction is also noticeable in the overall slope of the normal component *H_z_* (Figure 5i,l,o). It is best recognized by systematically rising field values at the sample edges. Compared to the elastic loading case (cf. Figure 5f), the transitions (white color) between both stray field directions (red and blue) are increasingly narrow, thus, causing larger *H_z_* gradients.

Opposed to *H_x_* and *H_z_*, the deformation-induced magnetization process is less apparent in the transverse in-plane component *H_y_* (Figure 5h,k,n). It is yet indicated by rising field values close to the specimen edges and in the direct specimen vicinity, respectively. Compared to the elastic loading case (Figure 5e), the number of *H_y_* stray field reversals noticeably increases with the extent of plastic deformation. 

In Figure 5g–o, local magnetic discontinuities are particularly striking, some of which are accentuated by grey arrows in the *H_z_* sub-images (Figure 5i,l,o). These localized distortions have similar *x*-*y* coordinates for each respective loading state and do not occur after elastic deformation. While they are sharply demarcated from their vicinity at the onset of microplastic deformation (specimen N2, Figure 5g–i), they progressively blur towards the specimen centers as the mechanical load increases (N3 and N4, Figure 5j–o). The two-dimensional data representation allows to spatialize these discontinuities and to distinguish for instance between elliptical and approximately straight-lined arrangements.

### 3.3. Strain Distribution

**Figure 6:**
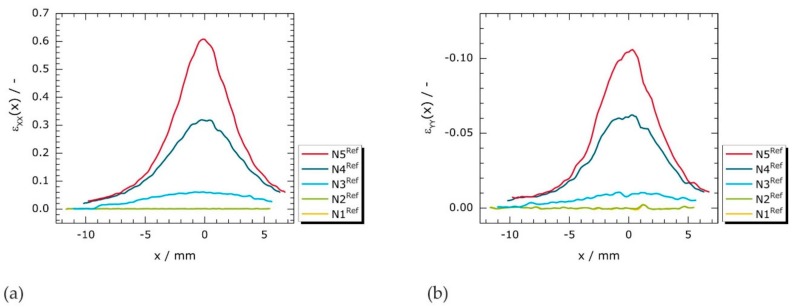
Measured strain profiles of reference loading stages N1^Ref^ to N5^Ref^ along *y* = 0: (**a**) longitudinal in-plane strains (*ε_xx_*(*x*)); and (**b**) transverse in-plane strains (*ε_yy_*(*x*)). Profiles extracted from 2D strain field measurements that are presented in Figure 7.

**Figure 7:**
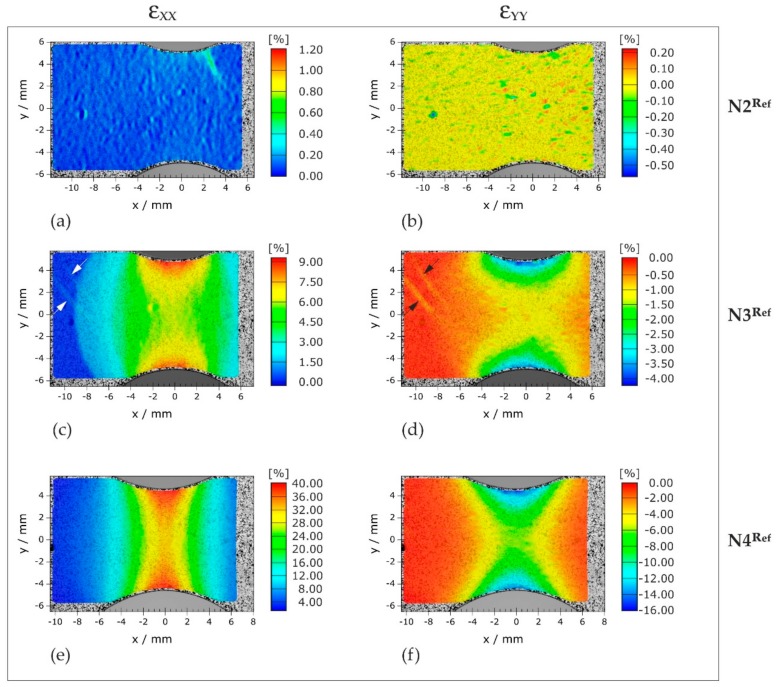
Strain fields measured optically under load (consecutive loading steps performed on one test piece): (**a**,**c**,**e**) longitudinal strains; and (**b**,**d**,**f**) transverse strains. Each row contains information about a specific loading stage, where N2^Ref^, N3^Ref^, and N4^Ref^ represent similar strain levels as N2, N3, and N4, respectively.

To the best of the authors’ knowledge, magnetic stray fields have not yet been correlated to experimentally determined strain fields in MMM. Therefore, spatially-resolved strain field measurements were performed on a reference specimen. Figure 6 shows profiles of longitudinal (*ε_xx_*(*x*)) and transverse (*ε_yy_*(*x*)) strains, respectively, that were extracted along *y* = 0 for each reference deformation state (N^Ref^) from the 2D strain fields shown in Figure 7. 

Along the horizontal symmetry axis (*y* = 0), differences between the curves of N1^Ref^ and N2^Ref^ are indeterminable in *ε_xx_*(*x*) (Figure 6a); the curves are superimposed. As the applied load increases, the progressive strain localization is clearly seen by peak-like curve progressions of N3^Ref^ to N5^Ref^. The amplitudes of the resulting peaks ascend with the applied mechanical load, whereas the *FWHM* values systematically decrease. In other words, the largest part of deformation is increasingly concentrated on an ever-smaller sample region. In principle, these observations are also valid for the transverse in-plane strains, *ε_yy_*(*x*) (Figure 6b). However, the material, which is longitudinally strained (positive *ε_xx_*(*x*) values), is compressed in transverse direction (negative *ε_yy_*(*x*) values). Note that the absolute values of *ε_yy_*(*x*) are approximately six times smaller than those of *ε_xx_*(*x*).

In Figure 7, the in-plane strain distributions in the notched sample areas of three reference deformation states are represented. In analogy to the magnetic field scans (Figure 5), longitudinal and transverse strains are arranged in columns, while the strains for a given deformation state are presented in rows. It is evident that longitudinal loading of notched specimens induces pronounced strain gradients and, hence, inhomogeneous (multiaxial) strain states. In fact, Figure 7a discloses locally elevated *ε_xx_* values (approx. by 0.5%) in the upper notch root at N2^Ref^ confirming the hypothesis that local yielding is already initiated in the very early and nominally elastic deformation stage N2. The fact that plastic deformation initially occurs in just one of the two notch roots hints at small inaccuracies in the alignment of the loading train. Starting from the notch roots, the plastically deformed regions propagate mainly in vertical (*y*) direction before also expanding from there in *x* direction (N3^Ref^, Figure 7c). In advanced loading stages (cf. N4^Ref^, Figure 7e), *ε_xx_* increasingly concentrates on a narrowing central region: while the *ε_xx_* values of N4^Ref^ rise noticeably in the region between the notches, no significant changes compared to N3^Ref^ (Figure 7c) are found at an *x* distance of approximately 9 mm to the center (dark blue).

The strain distribution of N3^Ref^ (Figure 7c) is particularly informative, since here the elliptical transition between the elastically (dark blue) and the plastically (light blue) deformed material area lies within the strain measurement field. Moreover, fine (Lüders) bands are recognizable (marked by arrows), which arise locally at the elastic-plastic transition and propagate from there towards the outside. The image suggests a band orientation of approximately 45° to the tangent of the elastic-plastic-transition line.

In principle, longitudinal straining (Figure 7a,c,e) is accompanied by contraction in *y* direction (Figure 7b,d,f). Nonetheless, Figure 7 reveals that the ratio of −*ε_yy_* to *ε_xx_* is not always constant and depends on the position. Keeping the example N3^Ref^ (Figure 7c,d), −*ε_yy_*/*ε_xx_* is approximately 0.4 in the notch root (*x* = 0, *y* = 4.1 mm), whereas it is approximately 0.15 at the center (*x* = *y* = 0). These differences are attributed to locally different amounts of constraint to transverse contraction because of evolving triaxial stress states. In other words, due to the notches, the material cannot contract transversely to the extent, which would result from the longitudinal strain levels in uniaxial loading cases. Note that in flat specimens, transverse contraction technically means a contraction in the *y* and in *z* directions (i.e., a reduction of width and of thickness). Hence, contraction in the *z* direction is also most likely locally constrained, which suggests an uneven reduction in the sample thickness.

### 3.4. Topography Evolution During Necking

**Figure 8:**
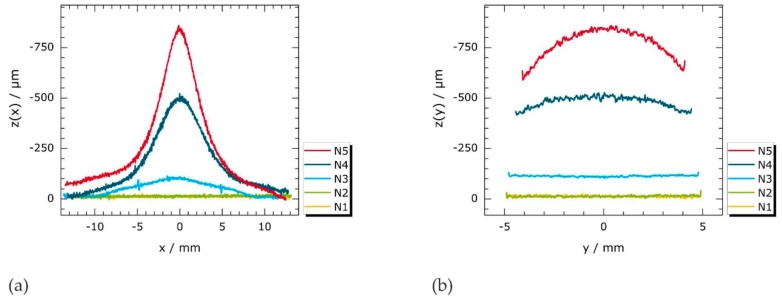
Topography (z) profiles of deformed specimens N1 to N5 measured by fringe projection (cf. Figure 9) disclose localized thickness reductions during necking. Note that *z* axes are inverted: (**a**) *z*(*x*) measured along *y* = 0; and (**b**) *z(y*) measured along *x* = 0.

**Figure 9:**
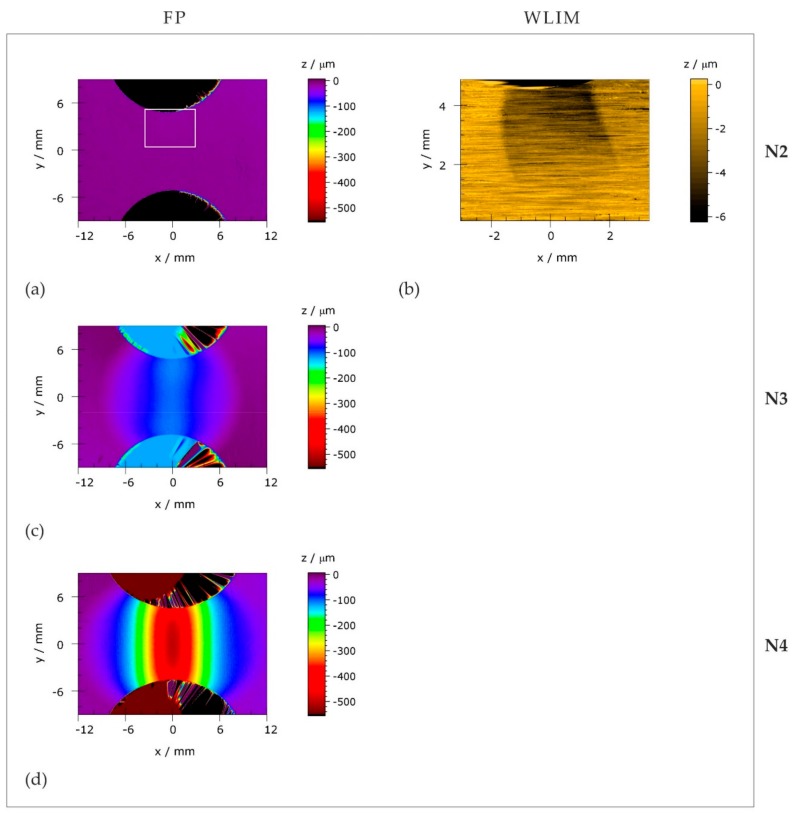
Surface topography resulting from nonlinear thickness reduction (necking): (**a**,**c**,**d**) Topography measured by fringe projection (FP) in notched regions of N2, N3, and N4, respectively; and (**b**) the white-light interference microscopy (WLIM) scan of N2 in the region marked by a white rectangle in (**a**).

Figure 8a,b present height profiles of N1 to N5 measured by fringe projection that were extracted along *x* = 0 and *y* = 0 from the 2D topography scans presented in Figure 9. A low background noise is apparent in all depicted curves, which is associated with the residual surface roughness caused by specimen grinding during the fabrication. 

The longitudinal height profiles (*z*(*x*), Figure 8a) of N1 and N2 tend to be linear and fall on a common line. In contrast, the sample thickness of N3 is reduced locally in the *x* range of ±9 mm; the lowest point of the sample surface (*z*(*x*) ≈ −100 μm) occurs close to the center (x ≈ 0). Moreover, artifacts appearing at *x* ≈ ±5 mm in this (N3) profile are striking, where the *z* values appear to escalate. These artifacts indicate the presence of topographic discontinuities (e.g., small steps or juts). As the applied load increases, pronounced (negative) peaks emerge in *z*(*x*). The amplitudes of these peaks successively grow, whereas the *FWHM* values continuously decrease. 

Figure 8b provides the height profiles along *x* = 0 (between the notch roots). In contrast to *z*(*x*) (Figure 8a), the sample thickness descends more uniformly along *x* = 0. In fact, the *z*(*y*) profiles of N1 to N3 are nearly linear; *z*(*y*) gradients develop only for very high applied mechanical loads (N4, N5).

The complete 2D topography scans of specimens N2 to N4 are presented in Figure 9. The measurement artifacts outside the notches, which are visible in Figure 9a,c,d, are caused by abrupt changes in height between the sample edges and the environment. Nevertheless, they do not affect the measurement results of the discussed surface regions.

It is hardly possible to account for first topographical changes at the surface of N2 using fringe projection technique, since the height differences here are within the range of the resolution limit. The complementary analysis method of white-light interference microscopy (WLIM) (Figure 9b), however, reveals that the sample thickness already decreases by a few microns when local yielding is initiated in the upper notch root of N2. For more advanced loading stages, both the sample width (*y* dimensions) and the thickness (*z* dimensions) are reduced (Figure 9c,d). As a result, the proceeding necking process induces a continuous decline of the sample cross-section.

Furthermore, two characteristic geometric features can be extracted from Figure 9c,d: first, the *z* gradients are larger in the *x* than in the *y* direction. Hence, the geometry of the necked region is comparable to that of a surface breaking crack, which takes a vertical course (i.e., it is running between the notches) and is greatly widened in the *x* direction. Second, elliptically-shaped transitions between necked and plane surface regions are indicated.

Finally, it should be noted that (assuming similar topographies on the back sides of the samples) the maximum thickness reductions of specimens N4 and N5 reach values in the order of 25 and 40 percent of the initial specimen thickness, respectively. Note that the resulting height differences in the sample surfaces are quite prominent and may change the local distance between the sample surface and the magnetic field sensor when measuring in constant height mode.

## 4. Discussion

### 4.1. The Role of Topography

It has been shown in Section 3.2 that weak magnetic stray fields are, indeed, detectable at the surfaces of the notched test pieces after longitudinal tensile loading. The results of the magnetic line measurements (Figure 4) and of the 2D distributions (Figure 5) both confirm that the magnetic state of the notched specimens systematically changes with increasing applied mechanical load. It is clearly seen that the three individually-measured magnetic field components differ significantly in terms of quality and quantity. In addition, the magnetic stray fields, the strain distributions (Section 3.3) and the surface topographies (Section 3.4) suggest complex interactions between magnetization, material mechanics, and geometrical effects.

The following qualitative similarities between surface magnetic fields, strains, and topographies are apparent: characteristic peaks exhibit in the individual profiles of H_x_*(*x*) (Figure 4a), *ε_xx_*(*x*) (Figure 6a), and *z*(*x*) (Figure 8a) after (local) plastic deformation with amplitudes systematically increasing with the applied mechanical load and global extreme values occurring at *x* ≈ 0. Moreover, the 2D data representations of H_x_* (Figure 5g,j,m), *ε_xx_* (Figure 7a,c,e), and *z* (Figure 9a,c,d) reveal that the magnitudes of *H_x_* in the necked region generally increase with higher longitudinal strain and lower sample thickness. In addition to these global observations, all Cartesian magnetic components show characteristic local discontinuities (e.g., marked by grey arrows in Figure 5l–o), such as elliptical structures at transitions between elastically and plastically deformed sample regions and straight-lined structures that correlate with individual Lüders bands. 

Nonetheless, there are also conspicuous differences: first, while *H_y_* and *H_x_* act in the same plane, neither the individual *H_y_* profiles nor the 2D distributions of *H_y_* indicate a straightforward correlation with transverse strains *ε_yy_*. It is only observed that direction reversals of *H_y_* occur within the necked specimen regions and near Lüders bands. Second, in contrast to the *ε_xx_*(*x*) and to the *z*(*x*) profiles (Figure 6a and Figure 8a, respectively), the *FWHM* values of H_x_*(*x*) systematically increase as the applied mechanical load rises, Figure 3a.

**Figure 10:**
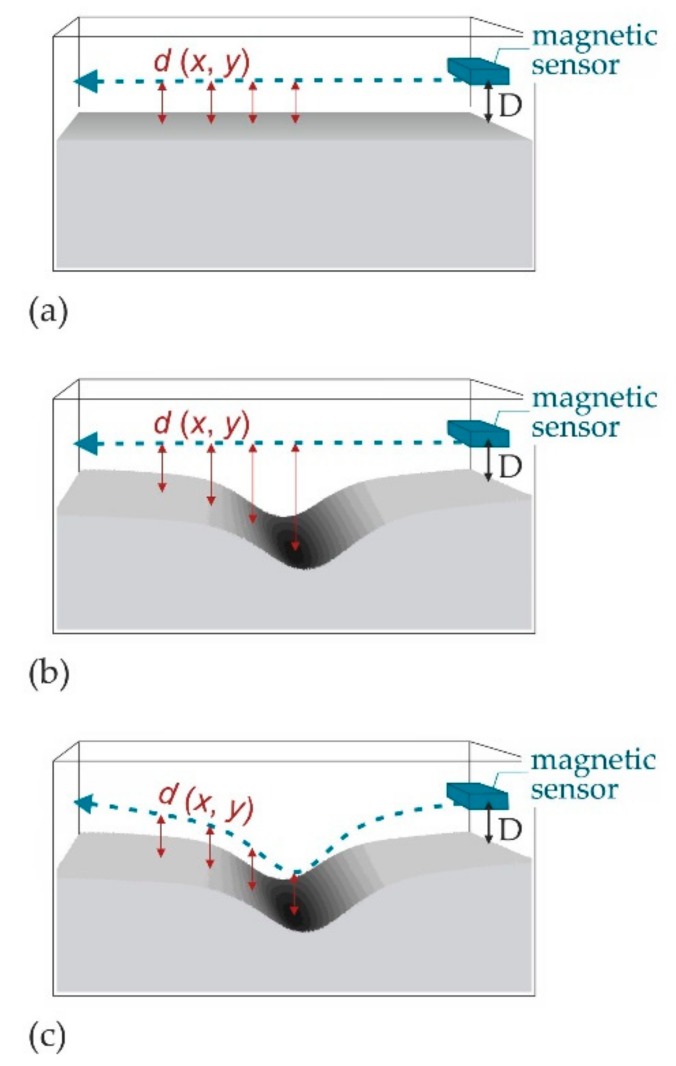
Illustration of topography-induced variances in the sensor to surface distance (lift-off, *d*): (**a**) lift-off and adjusted sensor height (*D*) are equal and constant for a level surface if scanned in constant height mode; (**b**) a topographic relief provokes position-dependent *d* values, when *D* remains constant; and (**c**) if the sensor height was variable (dotted blue line) during the measurement, constant *d* values would be achieved (idealized).

One reason for these dissimilarities may be the varying distance between the GMR sensor and the sample surface (lift-off, *d*) during the magnetic measurements, which results from local reductions of the sample thickness. In Figure 10, such lift-off variances caused by surface reliefs are represented schematically: if a flat, level surface is measured magnetically at a fixed, adjusted sensor height, *D*, the distance between the sensor and surface (lift-off, *d*) is always constant and equal to *D* (Figure 10a). The measured field strength, *H*, depends strongly on the distance between sensor and magnetic source (surface), with measured absolute field values decreasing with increasing distance to the source. If the surface to be measured exhibits topographical features, a constant sensor height will cause variable, position-dependent *d* values (Figure 10b), which, to the best of the authors’ knowledge, has not yet been regarded in previous MMM studies. Since the magnetic field distributions presented in Figure 4 and Figure 5 were measured at a fixed GMR sensor height, *D*, local falsifications of the magnetic signals due to variable *d* values must be assumed. 

Several authors propose the in-plane (often referred to as tangential) field component to be suitable for qualitative and quantitative assessments of a component’s stress state [13,28,31,32,33]. Thus, if the magnetic field strengths correlated with stresses, then the disregard of topography and variable sensors to surface distances could, especially for pronounced surface reliefs, lead to an underestimation of the actual stress state. Therefore, the measured magnetic field profiles H_x_*(*x*) and H_y_*(*x*) (Figure 4a,b) must be corrected with respect to their actual, variable *d*(*x*, *y*) values (cf. Figure 10b). In magnetostatics, the magnetic field (induced by a steady current) at a spatial distance *r* is usually calculated by the Biot-Savart law, where the magnetic field strength is inversely proportional to *r^n^* (with 1 < *n* < 3) [47]. To estimate the signal profiles for a constant (ideal) distance between sensor and surface *d*(*x*, *y*) = *D* (as depicted in Figure 10c), H_x_*(*x*) and H_y_*(*x*) are corrected using the Biot-Savart law conservatively with *n* = 1 as follows: 

The measured field strength H* is assumed to be inversely proportional to the actual lift-off, *d*(*x*, *y*) (cf. Figure 10b):

(2) $$H^{*}\propto \frac{1}{d\left(x,y\right)}$$

and for field strengths H** (corrected for a constant lift-off *d*, which shall be equal to *D* (cf. Figure 10c)), the following applies analogously:

(3) $$H^{**}\propto \frac{1}{D}$$

thus:

(4) $$\frac{H^{*}}{H^{**}}=\frac{D}{d\left(x,y\right)}.$$

Since the thickness reductions *z*(*x*, *y*) shown in Figure 8 and Figure 9 are defined as negative, the actual, position-dependent lift-off *d*(*x*, *y*) may be determined using the measured topography *z*(*x*, *y*) represented in Figure 8:

(5) d(x,y)=D−z(x,y),

which leads to the approximate solution of $H^{**}$:

(6) $$H^{**}=H^{*}\times \left(\frac{D-z\left(x,y\right)}{D}\right).$$

**Figure 11:**
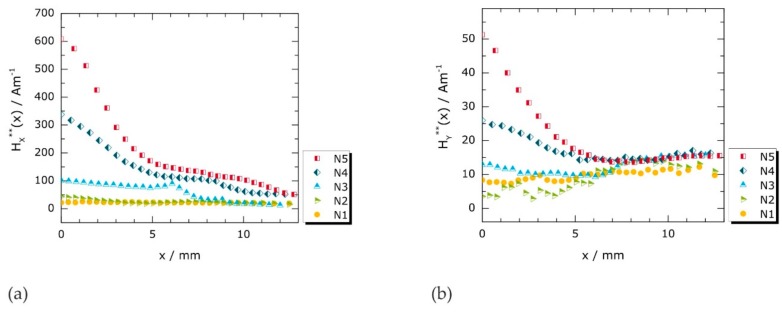
Lift-off corrected magnetic field profiles: (**a**) longitudinal component H_x_**(*x*); (**b**) transverse component H_y_**(*x*). Lift-off correction by application of Equation (6) using the magnetic data shown in Figure 4a,b and topography data from Figure 8a in the range of 0 < *x* < 13 mm.

Figure 11 shows the lift-off corrected field values H_x_**(*x*) and H_y_**(*x*) for one half of the notch zone of the specimens (0 < *x* < 13 mm). Note that the algebraic signs are of minor importance since they depend on the magnetic coordinate system defined. The lift-off correction for the left specimen halves would accordingly amplify negative field values. It is clearly seen that the lift-off correction causes both qualitative and quantitative changes in the signals compared to the measured H_x_*(*x*) and H_y_*(*x*) curves (cf. Figure 4a,b): opposed to the data obtained from measurements assuming a constant sensor height, not only the longitudinal component H_x_**(*x*), but also the transverse in-plane component H_y_**(*x*) tends to show peak curves with amplitudes being larger, the higher the applied mechanical load. Since the H_y_* values are generally much lower than the H_x_* values, the lift-off correction only slightly enhances the sensor noise for the case of N1 to N3. Therefore, H_y_** fluctuations of the curves N1 to N3 in Figure 11b should not be over-interpreted. However, for advanced stages of deformation, the maximum values of both H_x_** and H_y_** (at *x* ≈ 0) noticeably exceed those of the measured curves (H_x_*(*x*) and H_y_*(*x*)). For example, they are about 1.9 times higher for N4 and about 2.4 times higher for N5 as compared to the uncorrected values. 

Since H** is a function of thickness reduction (Equation (6)) and the *z* values are, in all curves, comparatively small for large |*x*| positions, the lift-off correction alters the original field values for large |*x*| positions (e.g., at *x* ≥ 10 mm) only marginally. As a result, the additional local extrema and inflection points observed in H_x_*(*x*) and H_y_*(*x*) (Figure 4) are less pronounced in the lift-off corrected curves presented in Figure 11. This particularly topography-dependent amplification of magnetic field strengths results in *FWHM* values, which now continuously decrease with increasing deformation in both in-plane field components. Consequently, a qualitatively good agreement of the lift-off corrected profiles H_x_**(*x*) and H_y_**(*x*) with the magnitudes of the *ε_xx_*(*x*) (Figure 6a) and *ε_yy_*(*x*) (Figure 6b) along the horizontal symmetry axis (*y* = 0) is achieved. Nonetheless, the measured H_y_* distributions (Figure 5h,k,l) clearly show magnetic stray fields with alternating field directions and peak values occurring outside the analyzed lines. Applying Equation (6) to H_y_* values for *x*-*y* coordinates beyond the axes of symmetry (e.g., along *y* = 3 mm) could, therefore, hardly bring about qualitative similarities with the corresponding transverse strains, *ε_yy_* (Figure 7b,d,f). 

Actually, the role of surface topography is not confined to potential changes in the lift-off value: as shown in Section 3.4, the sample thickness reduces progressively and increasingly localizes as the applied mechanical load rises. During the necking process, the ratio of thickness reduction, -*z* to the width (*x* dimension) of the necked region increases gradually (cf. Figure 9). In other words, the geometry of the necked region more and more resembles that of a flat and wide surface breaking crack (running in transverse (*y*) direction). 

The detection of cracks by established magnetic NDT techniques, such as magnetic flux leakage (MFL) testing, is based on the analysis of magnetic stray fields that arise at geometric flaws (e.g., gaps), when an external magnetic field is applied [48,49]. Although components to be investigated by MFL testing are usually magnetized to magnetic saturation, the signals should be qualitatively comparable to those forming under very weak external fields (e.g., the Earth’s magnetic field).

**Figure 12:**
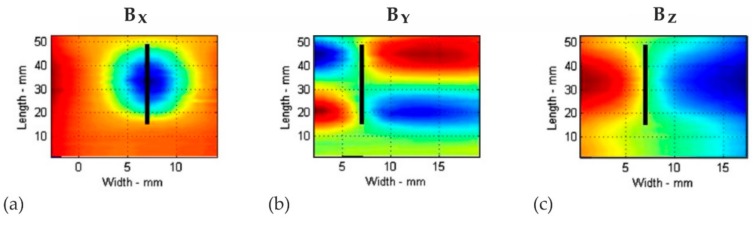
Magnetic stray field formation around an artificial crack (indicated by solid black lines in all sub images). Reused from [50], Copyright (2007), with permission from Elsevier; 3D magnetic flux leakage (MFL) measurement of test piece magnetized to saturation in the longitudinal (width) direction; (**a**) in-plane component ∥ to width and ⊥ to crack, *B_x_*; (**b**) in-plane component ⊥ to width and ∥ to crack, *B_y_*; and (**c**) component ⊥ to the surface, *B_z_*.

The 3D magnetic flux density distributions (*B*) around an artificial surface crack determined by Li, et al. are represented in Figure 12 [50]. They were recorded using a three-axis anisotropic magneto-resistive (AMR) sensor after a slotted steel plate had been intentionally magnetized in *x* direction. The magnetic flux *B* is linked to the magnetic field strength *H* via the permeability of free space µ_0_, where *B* = µ_0_*H*. While the quantitative *B* values are not specified by Li et al. [50] and, as mentioned before, the algebraic signs depend on the magnetic sensor coordinate system, the *B_x_*, *B_y_*, and *B_z_* distributions in Figure 12 show striking qualitative similarities to the measured *H_x_*, *H_y_*, and *H_z_* distributions presented in Figure 5g–o: in the viewing plane, *B_x_* (acting transversely to the crack, Figure 12a) exhibits stray fields, which are characterized by an elliptical flux distribution and extreme values at the center of the geometrical defect. *B_y_* clearly shows four regions of alternating field directions that are separated by a horizontal and by a vertical axis of symmetry (Figure 12b) and the normal component *B_z_* possesses two large extrema left and right of the crack (Figure 12c). 

The similarity between *B_y_* (Figure 12b) and the measured *H_y_* distributions (Figure 5h,k,l) suggests that, particularly, the *H_y_* component reflects the geometry of the necked region rather than the (transverse) strain distribution (Figure 6b,d,f). Consequently, the emerging topography (acting as a geometric discontinuity) must be causally involved in the formation of magnetic stray fields. Additionally, the presence of local magnetic discontinuities in *H_x_*, *H_y_*, and *H_z_* (Figure 4g–o) that are observed only after the onset of microplastic deformation (e.g., at positions of the Lüders bands) supports this assumption: using high-resolution WLIM (Figure 8b), first, topographical changes resulting from local yielding could be detected at positions where local discontinuities first appear in *H_x_*, *H_y_*, and *H_z_* (Figure 4g,h,i). 

The MMM technique is a standardized NDT technique [25]. Several authors state that quantitative information about a component’s damage or stress state can be derived from the magnetic stray fields [13,14,26,29]. Yet, the physical origins of the magnetic stray field formation have so far mainly been attributed to (i) macroscopic stress gradients in the material (often referred to as “stress concentration zones”, SCZ) [13,17,18,19,26,29]; (ii) (locally) increased dislocation densities [13,16,18,19,26]; and (iii) the associated local magnetic permeability reduction due to plastic deformation [14,17,29]. Note that topography must be expected for most of the damage states being within the scope of MMM testing, such as welded joints (seam topography) [51,52,53], bulging, buckling, and barreling under non-uniform compressive loading [54,55,56], corrosion pits and stress corrosion cracking [57,58,59,60], inhomogeneous growth and spalling of (e.g., iron) oxide scales [61], and slip bands, and even intrusions and extrusions resulting from fatigue loading [62,63,64,65,66]. Our results demonstrate that disregarded surface topographies may provoke safety-relevant misinterpretations when using the MMM technique for quantitative stress analyses. Therefore, to account for potential topographical contributions, (i) the orientation of individually analyzed magnetic field components with respect to the defect geometry should be known; (ii) all three individual magnetic vector components should be recorded and analyzed; (iii) the surface topography should be measured simultaneously; and (iv) a variable sensor lift-off should be considered.

### 4.2. Multiaxiality

Based on the results discussed, it is not possible to determine with certainty whether the magnetic stray fields arise solely as a result of the mentioned topographic/geometric changes or due to a superposition of geometrical and magnetomechanical effects. However, when magnetic stray fields resulting from inhomogeneous elastic-plastic deformation are to be correlated with mechanical quantities, such as stresses or strains, some general considerations arise.

Stress concentration, localized straining and damage processes in general are associated with multiaxial stress and strain states in the material. However, multiaxiality is often overlooked or insufficiently considered in MMM publications. Since stress and strain (Figure 7) gradients form under multiaxial loading conditions and the actual stress/strain values are position-dependent, correlations of local magnetization values with nominal stresses/strains (such as [13,16,17,18,20,23,28,67]) do not seem appropriate. Note that both local and global stress/strain values are not only dependent on the material investigated and the applied mechanical load, but also on the geometry (shape and size) of the component, specimen, or notch used. Moreover, the geometry is progressively altered by plastic deformation.

Figure 5 clearly shows that the individual *H* components provide directional information. Therefore, also a spatially-resolved correlation of individual magnetic vector components with scalar (directionless) equivalent stresses (e.g., as performed by Roskosz et al. [14,68]) is not self-explanatory. Since magnetomechanical interactions have mainly been studied and explained for uniaxial loading conditions, it can only be assumed that (directional) principal stresses/strains could be more favorable quantities for potential correlations with individual magnetic field components. However, in contrast to the measured vector directions of *H_x_*, *H_y_*, and *H_z_* (usually being invariant during magnetic measurements), the mechanical principal directions may rotate due to complex specimen geometries (e.g., in the direct vicinity of notches). In other words, potential correlations of individual magnetic components with principal stresses/strains may then require position-dependent rotations of the magnetic field vector with respect to variable principal stress/strain axes.

Further, when explaining the formation of magnetic stray fields in the presence of stress concentration, the MMM literature usually refers to the inverse effect of magnetostriction discovered by Villari in 1865 [1], which is also called the magnetoelastic (Villari) effect [69] or the magnetomechanical effect [45]: after Joule had reported on detectable changes in the dimensions (i.e., strains) of iron wires and bars under external magnetization (known as magnetostriction) [70], Villari observed magnetic changes in iron and steel during mechanical tensile loading. It is worth noting that, although Villari did not explicitly refer to forces or stresses but rather to the tensile loading process [1], the assumption of magnetoelasticity being a stress-controlled process has prevailed in the MMM literature [13,14,15,16,17,18,19,20,21]. Since stresses and strains, according to Hooke’s law, change proportionally during uniaxial elastic deformation, they may easily be transferred into one another based on geometric considerations using the elastic constants (e.g., Young’s modulus, *E*, and Poisson ratio, *ν*). It may, therefore, be of minor importance whether magnetic changes are responses to applied elastic stresses [7,71,72] or elastic strains (induced by applied stresses) [12]. However, for plastic deformation, where the uniaxial strain response to a uniaxial stress increment is larger than for elastic deformation (cf., e.g., Figure 3) and the uniaxial stress-strain behavior is not proportional, the mechanical field size characterizing amount and direction of magnetization should be explicitly indicated. 

Whether magnetic polarization in a small, constant magnetic field obeys rather to stress than to strain changes may be particularly important for multiaxial deformation and in the presence of hydrostatic stress parts. For example, if the principal stresses acting on a material volume are compressive in all three directions but unequal in magnitude, the material may, yet, be (positively) strained in one principal direction. In this study, the stray fields evolving during inhomogeneous plastic deformation (discussed in Figure 11) show remarkable analogies to experimentally determined strains (Figure 6). It may, therefore, be worth investigating whether magnetic correlations with strains may offer advantages over correlations with stresses, when a ferromagnetic material is loaded homogeneously or inhomogeneously within the plastic range. 

Plastic deformation is accompanied by irreversible changes in the microstructure, which may strongly affect magnetic features. For example, a high dislocation density increases the coercive force and decreases remanence and permeability. Highly plasticized material regions, hence, become magnetically harder than other material regions [73,74]. Due to these position-dependent permeability changes, magnetic stray fields may arise, wherein the absolute values depend greatly on internal magnetic fields. However, although dislocations and their interactions with domain walls (simply referred to as the “pinning effect”) are assumed to be the microstructural cause of the formation of magnetic stray fields [13,16,18,19,26], correlations of magnetic stray fields with experimentally determined and statistically relevant dislocation densities are missing in MMM studies. Moreover, from a microstructural point of view, plastic deformation and early damage of ductile metals are not only associated with slipping, pile-up and multiplication of dislocations (strain hardening). For large plastic deformations, local deformation textures [75,76,77,78] may also arise and, thus, may cause local accumulations of magnetically harder or softer directions, i.e., cause local magnetocrystalline anisotropies. In addition, formation, growth and coalescence of voids precede microscopic and macroscopic crack initiation [79,80,81]. While it is very well conceivable that both local crystallographic textures resulting from inhomogeneous plastic deformation and voids may also contribute to the permeability reduction reported in [14,17,29], the role of such location-dependent microstructural changes has, to the best of the authors’ knowledge, so far neither been investigated nor considered in the MMM literature. 

Finally, it is worth noting that, irrespective of the actual mechanical or microstructural origins, such a macroscopic magnetic effect must be achieved by systematic changes in the magnetic microstructure (i.e., the magnetic domain structure). In contrast to, e.g., Fe-Si transformer steels tailored to their magnetic properties, structural steels are usually fine-grained, non-oriented (i.e., not textured specifically for optimum magnetic properties) and subject to only moderate specifications regarding chemical composition and permissible heat treatment states. Magnetic domain configurations in structural steels are, therefore, more strongly influenced by the microstructure (e.g., crystallographic orientations of individual crystallites) and are already very complex in the manufacturing state. 

As described, macroscopic multiaxial deformation may lead to principal stress and strain directions locally differing from the applied stress/strain coordinate system. It also induces short- and long-range stress and strain gradients that may cause some regions in the material to be elastically deformed while others plasticize. Moreover, within the plasticized regions, dislocation densities may vary, and severely deformed grains may rotate and alter magnetocrystalline anisotropies. Consequently, magnetic domains are presumably differently affected by different variables in different material regions under inhomogeneous, elastic-plastic deformation. To understand these changes in the overall magnetic domain distribution, experimental investigations including magnetic domain imaging at the mesoscale seems essential. Such attempts have, to the best of the authors’ knowledge, so far also only rarely been made [82] and should be further pursued to obtain a better understanding of the underlying macro- and micro-mechanisms. 

From all these considerations it follows that the physical causes of the magnetic stray field formation during localized (multiaxial) elastic-plastic deformation still appear insufficiently understood both at the macroscopic and at the microscopic scale. In addition, the more general issue of a proper correlation of directional magnetic field components with mechanical tensors (or components) with due regard to multiaxial stress/strain states should be further investigated and discussed. Consequently, a quantitative stress assessment based on residual magnetic stray fields currently seems premature. 

## 5. Conclusions

Macroscopic residual magnetic stray fields resulting from inhomogeneous elastic-plastic deformation of a medium-soft ferromagnetic material (exemplified by an unalloyed structural steel) have been investigated by analyzing the results of the spatially-resolved magnetic field, strain field, and topography measurements, respectively. The following conclusions can be drawn:

- For the analysis and correlation of gradients (in, e.g., magnetic fields, stresses, strains), spatially-resolved measurement methods and two-dimensional data representations should be preferred over global examination methods and the analysis of individual profiles. They may reveal specific geometric features of characteristic structures (e.g., Lüders bands), particularly, when the (magnetic) changes are too small to be reliably distinguished from the measurement noise of the (magnetic) sensor used.
- Topographic changes due to localized plastic straining act as geometric discontinuities and must be considered as one of the basic causes for the observed magnetic stray field formation.
- Topographic changes may lead to variable distances between magnetic sensing device and analyzed specimens or component surfaces (lift-off). Since recorded magnetic intensities depend on the distance to the magnetic source, a variable lift-off may distort the magnetic signals qualitatively and quantitatively and, thus, may provoke misinterpretations.
- Therefore, quantitative stress or damage assessments on the basis of residual magnetic field distributions of inhomogeneously (plastically) deformed surfaces should be avoided until the magnetomechanical interrelations and the underlying mechanism of the stray field formation are sufficiently well understood. To this end, true multidisciplinary and multi-scale research is still required, for instance regarding:
  (i) options for the computational correction of topography-related artefacts;
  (ii) the correlation of magnetic field vectors (and their components) and stress-strain tensors (and their components);
  (iii) the role of locally emerging deformation textures with respect to local changes in magnetocrystalline anisotropy;
  (iv) the role of formation, growth and coalescence of voids during the ductile damage process and their influence on macroscopic magnetic quantities such as permeability, coercivity, saturation magnetization, and hysteresis loss; and
  (v) microscopic and macroscopic (statistical) changes in the magnetic domain structure (of polycrystalline ferromagnetic materials) caused by complex states of stress and strain.

## Figures and Tables

**Table 1:** Chemical composition of S235JR determined by optical emission spectrometry (OES); averaged from five measurements.

Element	C	Si	Mn	P	S	Cr	Cu	Mo	Nb	Ni	Ti	V	Fe
mass%	0.14	0.012	0.52	0.011	0.008	0.027	0.034	0.004	0.003	0.017	<0.001	0.004	99.1

**Table 2:** Mechanical properties of S235JR determined on smooth, flat standard test piece in monotonic tension.

Young’s Modulus, *E* in GPa	Upper Yield Strength, *R_eH_* in MPa	Strain at *R_eH_* in %	Lower Yield Strength, *R_eL_* in MPa	Strain at *R_eL_* in %	Ultimate Tensile Strength, *UTS* in MPa	Strain at *UTS* in %
205.6	256	0.16	228	1.92	375	22.55

**Table 3:** Nominal stress (*σ*_n_) and strain (*ε*_n_) limits of specimens N1 to N5.

Specimen	N1	N2	N3	N4	N5
*σ*_n_ in MPa	103	201	365	403	369
*ε*_n_ in %	0.05	0.07	2.68	9.66	13.56

**Figure A1:**
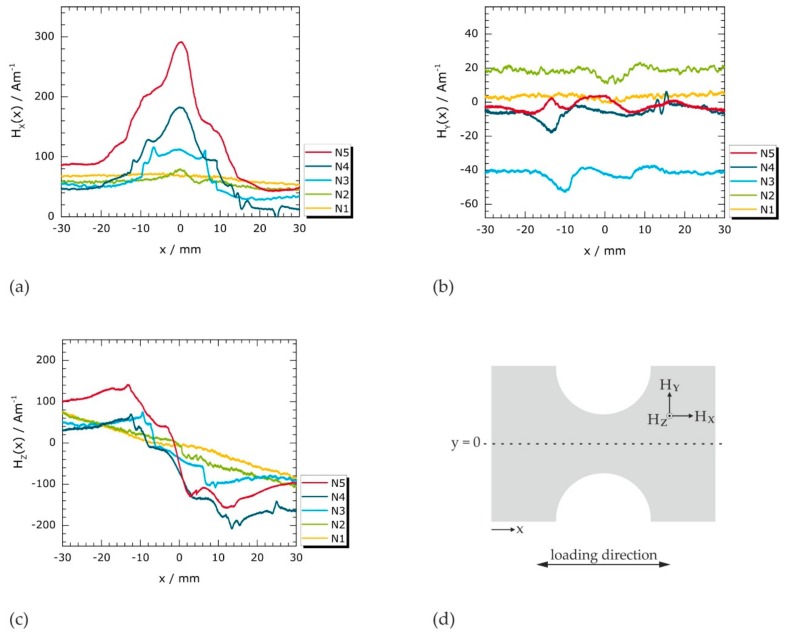
Original (measured) GMR data: (**a**) longitudinal in-plane field component *H_x_*(*x*), (**b**) transverse in-plane component *H_y_*(*x*), and (**c**) normal component *H_z_*(*x*) measured at the surface along *y* = 0 as depicted in (**d**).

**Figure A2:**
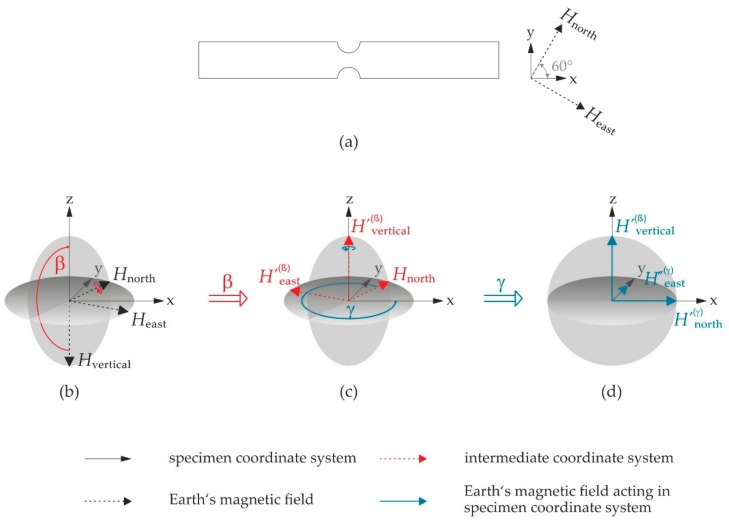
Approximation of Earth’s magnetic field (EMF) contributions acting in the directions of the specimen coordinate system: (**a**) specimen positioning with respect to geographic north and east; (**b**–**d**) coordinate transformation EMF vector in two steps. Rotation around *H_north_* axis by β = 180° (**b**) results in intermediate coordinate system (**c**), which is then rotated around *H’_vertical_* axis by γ = 300° to become coaxial with the specimen coordinate system (**d**).

## Appendix A

The *H** curves presented in Figure 4 are offset-corrected (cf. Appendix B). Figure A1 provides the original data of *H_x_*, *H_y_* and *H_z_* in the *x* range of ± *x* = 30 mm (along *y* = 0).

## Appendix B

The calibration of the used GMR magnetometer (prototype) is challenging for several reasons: first, the “zero point” of the sensor is unknown because there are no suitable reference samples available for calibration in the weak geomagnetic field. Second, unlike gradiometers, magnetometers measure absolute field values so that influences of unknown or known surrounding, static, or dynamic magnetic fields (e.g., produced by the small motors of the measuring table) can never be safely excluded. The same applies to absolute measurements of magnetic field strength during mechanical loading. Therefore, the offset values of the magnetic field matrices were estimated using the Earth’s magnetic field components as described below. Note that we also provide the original data in Appendix A.

### Appendix B1. Specimen Excitation by Earth’s Magnetic Field During Magnetic Measurements

The measurements were performed in April 2015 in Berlin, Germany. Intensities of the International Geomagnetic Reference Field (IGRF) were determined via an IGRF declination calculator [83]:

north component: 18,646.2 nT (corresponds to 14.88 A/m)

east component: 1125.3 nT (corresponds to 0.89 A/m)

vertical component: 45,898.3 nT (corresponds to 36.49 A/m)

To deduce the Earth’s magnetic field (EMF) intensities acting in the specimen coordinate system (SCS), the EMF vector was transferred into SCS by two rotation steps as shown in Figure A2b–d. The rotated components of EMF are:

*H’_north_* = *H_x_^EMF^* = 8.21 A/m

*H’’_east_* = *H_y_^EMF^* = 12.44 A/m

*H’_vertical_* = *H_z_^EMF^* = −36.49 A/m

### Appendix B2. Offset-Correction

For numerical purposes, the EMF was not compensated (zero-balanced). Instead *H’_north_*, *H’’_east_*, and *H’_vertical_*, respectively, were used for the offset-correction of *H_x_*(*x*), *H_y_*(*x*), and *H_z_*(*x*) (cf. Figure A1 and Figure 4): magnetic field strengths of the in-plane components (*H_x_*, *H_y_*) were assumed to be in the order of *H’_north_* and *H’’_east_*, respectively, when measured far away from the notched region (where plastic deformation processes can safely be excluded for all loading stages). Therefore, the differences between *H_x_*, *H_y_* and *H’_north_*, *H’’_east_* were determined at *x* = +30 mm, *y* = 0 for each specimen and the curves were then shifted with respect to their specimen-specific offset values. The offset-corrected curves are asterisked in Section 3.2.1.

It was further assumed that *H_z_* was in the order of *H’_vertical_* at the position of the polarity reversal (*x* = *y* = 0). Hence, the *H_z_*(*x*) curves were corrected in such a way that *H_z_**(*x* = y = 0) became equal to *H’_vertical_*.

The field values discussed in Figure 4, Figure 5 and Figure A1 were recorded by the sensor scanning from the right to the left image sides. While the spatial resolution of the three-axis GMR magnetometer surpasses commercially marketed MMM sensors, a known shortcoming of GMR sensors is their affinity to hysteresis effects [84]: the GMR sensors, especially when passing the notches, drag their measuring histories into the left half of the images. Therefore, both offset correction and lift-off correction (Section 4.1) were conducted for positive *x* values.
